# Neuropathic Injury–Induced Plasticity of GABAergic System in Peripheral Sensory Ganglia

**DOI:** 10.3389/fphar.2021.702218

**Published:** 2021-07-27

**Authors:** Caixue Wang, Han Hao, Kaitong He, Yating An, Zeyao Pu, Nikita Gamper, Hailin Zhang, Xiaona Du

**Affiliations:** ^1^ The Key Laboratory of Neural and Vascular Biology, The Key Laboratory of New Drug Pharmacology and Toxicology, Department of Pharmacology, Ministry of Education, Hebei Medical University, Shijiazhuang, China; ^2^ Faculty of Biological Sciences, University of Leeds, Leeds, United Kingdom

**Keywords:** GABA_A_ channel, DRG, neuropathic pain, α5 subunit, plasticity

## Abstract

GABA is a major inhibitory neurotransmitter in the mammalian central nervous system (CNS). Inhibitory GABA_A_ channel circuits in the dorsal spinal cord are the gatekeepers of the nociceptive input from the periphery to the CNS. Weakening of these spinal inhibitory mechanisms is a hallmark of chronic pain. Yet, recent studies have suggested the existence of an earlier GABAergic “gate” within the peripheral sensory ganglia. In this study, we performed systematic investigation of plastic changes of the GABA-related proteins in the dorsal root ganglion (DRG) in the process of neuropathic pain development. We found that chronic constriction injury (CCI) induced general downregulation of most GABA_A_ channel subunits and the GABA-producing enzyme, glutamate decarboxylase, consistent with the weakening of the GABAergic inhibition at the periphery. Strikingly, the α5 GABA_A_ subunit was consistently upregulated. Knock-down of the α5 subunit *in vivo* moderately alleviated neuropathic hyperalgesia. Our findings suggest that while the development of neuropathic pain is generally accompanied by weakening of the peripheral GABAergic system, the α5 GABA_A_ subunit may have a unique pro-algesic role and, hence, might represent a new therapeutic target.

## Introduction

The inhibitory GABAergic and glycinergic interneurons are crucial components of the “gate” on the way of nociceptive input to the spinal cord, proposed over 50 years ago in the gate control theory of pain (Melzack and Wall, 1965). These interneurons conduct pre- or postsynaptic inhibition with regard to excitatory interneurons, projection neurons, and primary afferent terminals and, thus, control the transmission of nociceptive information from the periphery (Braz et al., 2014; Duan et al., 2014; Mendell, 2014; Peirs et al., 2015; Zhang et al., 2018).

GABA is an important inhibitory neurotransmitter in the mammalian central nervous system (CNS). Its inhibitory effects are mediated *via* anion-selective ionotropic GABA_A_ receptors and metabotropic GABA_B_ receptors (Kumar et al., 2013; Ito, 2016). The activation of GABA_A_ receptors in most adult CNS neurons leads to an influx of Cl^−^, resulting in membrane hyperpolarization and suppression of neuronal excitability. In contrast to the central nervous system, GABA_A_ receptor activation in the primary sensory neurons causes primary afferent depolarization (PAD) (Price et al., 2009; Wilke et al., 2020). GABA_A_-mediated depolarization occurs due to the accumulation of high intracellular Cl^−^ concentrations in peripheral sensory neurons because of the high expression of the Cl^−^-importing transporter, NKCC1, relative to the expression of the Cl^−^-extruding transporter, KCC2 (Coull et al., 2003; Pitcher and Cervero, 2010). PAD inhibits peripheral input to the spinal cord, mainly due to the shunting effect of GABA_A_ Cl^−^ conductance, resulting in a reduction in the incoming spike amplitude and presynaptic Ca^2+^ influx, and subsequently reduced neurotransmitter release from presynaptic terminals of peripheral afferents (Watson et al., 2005). However, in certain pathological situations, due to the increase in NKCC1 activity and further [Cl^−^]_i_ accumulation, the GABA-induced depolarization may drive the membrane potential toward the action potential threshold, resulting in a dorsal root reflex (DRR) (Willis, 1999; Price et al., 2009).

Although it is generally accepted that the spinal dorsal horn is the first point of integration of pain signals within the somatosensory pathways, growing evidence has suggested that primary sensory neurons can also influence sensory transmission in the manner of an additional “gate” or “filter” (Dun, 1955; Stoney, 1990; Gemes et al., 2013; Du et al., 2014; Du et al., 2017). Thus, we recently demonstrated that dorsal root ganglion (DRG) neurons expressed proteins necessary for GABA synthesis, transport, and release, as well as multiple GABA receptor subunits (Du et al., 2017). DRG neurons are pseudounipolar, that is, they emit single axons that bifurcate into central and peripheral branches; somatic GABA_A_ receptor activation in DRG neurons inhibits nociceptive transmission through the ganglion mainly by increasing the rate of spike failure to propagate through the axonal bifurcation (*t*-junction). Pharmacological, chemogenetic, or optogenetic stimulation of the GABAergic system within the DRG *in vivo* strongly alleviated physiological (nociceptive), inflammatory, and neuropathic pain (Du et al., 2017).

Reduction or elimination of spinal cord inhibitory circuits is reported to play a role in generating chronic pain states (Sugimoto et al., 1990; Moore et al., 2002; Lever et al., 2003; Scholz et al., 2005; Etlin et al., 2016; Llewellyn-Smith et al., 2018). For instance, downregulation of the GABA-synthesizing enzyme (Moore et al., 2002), pre- and postsynaptic GABA receptors (Polgar and Todd, 2008), or dysregulation of Cl^−^ homeostasis (Chen et al., 2014; Modol et al., 2014) in the spinal dorsal horn are important contributors to the generating of neuropathic pain. Moreover, restoration of the spinal GABAergic inhibitory system by implantation of GABAergic progenitor cells provides long-lasting pain relief (Etlin et al., 2016; Llewellyn-Smith et al., 2018). Although there are some relevant reports, our understanding of chronic pain–associated plasticity within the peripheral GABAergic system is still incomplete (Pathirathna et al., 2005; Price et al., 2009; Zhu et al., 2012; Zhang et al., 2015; Loeza-Alcocer et al., 2019). Therefore, in this study, we aimed to examine the changes in the functional expression of GABA-related proteins in DRG neurons during the development of neuropathic pain induced by chronic constriction injury (CCI) of the sciatic nerve.

## Materials and Methods

**Animals.** Adult male Sprague Dawley rats (180–200 g) were used in this study. All animal experiments were approved by the Animal Care and Ethical Committee of Hebei Medical University (Shijiazhuang, China) and were in accordance with the International Association for the Study of Pain guidelines for animal use. The ethical approval reference number is IACUC-Hebmu-2020007.

**Neuronal cultures.** DRG neurons were dissociated and cultured as described previously (Linley et al., 2008; Liu et al., 2010). Briefly, adult rats were humanely euthanized by cervical dislocation under isoflurane anesthesia. Rats were decapitated, and the spines were removed and sectioned longitudinally. L4-6 DRGs were removed and dissociated, respectively, in Hank’s Balanced Salt Solution supplemented with 1 mg/ml type 1A collagenase (Sigma-Aldrich) and 10 mg/ml dispase (Invitrogen) in a humidified incubator at 37°C and 5% CO_2_ for 30 min. Ganglia were then gently triturated, washed twice by centrifugation, and resuspended in 600 μl culturing medium; this suspension was then plated onto 10-mm glass coverslips pre-coated with poly-d-lysine and laminin. Neurons were left to attach to the coverslips for 4–8 h in a humidified incubator (37°C, 5% CO_2_) and were recorded straight after.

**Electrophysiology.** Gramicidin-perforated patch clamp recordings were used to record GABA currents from DRG neurons at room temperature. Patch pipettes (with a resistance of 2–4 MΩ) were fabricated from borosilicate glass capillaries using a Sutter P-97 puller (Sutter) and fire-polished. Currents were amplified and recorded using an Axon MultiClamp 700B amplifier and pClamp 10.4 software (Axon Instruments) and were sampled at a frequency of 5 kHz. GABA currents from DRG neurons were recorded at a holding potential of −60 mV. The bath solution contained the following (in mM): NaCl (145), KCl (5), CaCl_2_ (2), MgCl_2_ (2), HEPES (10), and glucose (10), with a pH value of 7.4, adjusted with NaOH. The pipette solution contained (in mM): KCl (150), MgCl_2_ (5), HEPES (10), Mg-ATP (3), and Na-GTP (0.6), supplemented with 400 μg/m gramicidin (Sigma), with a pH value of 7.4, adjusted with KOH.

**Quantitative real-time RT-PCR.** Total RNA from the combined L4–L6 DRGs of an adult rat with CCI or a sham-operated control rat was extracted using a commercial RNA isolation kit at 5 or 14 days after operation (RNAiso, Takara). Isolated RNA was dissolved in 20 μl diethyl pyrocarbonate–treated (DEPC-treated) water and reverse-transcribed using a PrimeScriptRT™ reagent kit (Takara) and a thermal cycler (Mastercycler, Eppendorf). Quantitative PCR reaction was performed using SYBR Premix Ex TaqII (Takara), and the fluorescent DNA was detected and quantified using an FQD-48A (A4) system (BIOER). A list of primers used in standard RT-PCR experiments is given in Supplementary Table S1.

**Western blotting.** The DRG lysates were prepared with RIPA lysis buffer and protein quantified using a BCA Protein Assay Kit (Thermo). About 20 µg of protein was dissolved in 5 × SDS sample buffer (Invitrogen) and separated on NuPAGE 8% Bis–Tris gels (Invitrogen) for 120 min at 80 mV. Protein bands were then electro-transferred to a polyvinylidene difluoride membrane (Thermo Scientific) for 150 min at 200 mA. Membranes were blocked with 5% nonfat milk/TBST for 1.5 h at room temperature and incubated with the primary antibody against the α5 GABA_A_ subunit (Abcam) and GAPDH (Proteintech) in 5% nonfat milk/TBST overnight at 4°C. The blots were visualized by enhanced chemiluminescence, and images were captured by using a Kodak Image Station 4000 R and quantified using Kodak MI SE software. GAPDH was used as the internal control.

**Immunocytochemistry.** DRGs were excised, submerged in Tissue-Tek O.C.T. (Sakura), frozen, and sectioned (8 μm) using a freezing microtome CM 1950 (Leica). DRG slices were fixed in 4% paraformaldehyde (PFA) for 15 min at room temperature (RT). Cells were washed three times with PBS and then permeabilized with 0.1% Triton X-100 for 10 min at RT. To block nonspecific antibody binding, cells were incubated with 10% goat serum in PBS (blocking buffer) for 30–60 min at RT. DRG slices were then incubated overnight with antibodies against α1 (Abcam, cat. no. ab252430, 1:100), α4 (Abcam, cat. no. ab242008, 1:100), α5 (Abcam, cat. no. ab259880, 1:200), and β2 (Abcam, cat. no. ab15600, 1:100) of GABA_A_ subunits, the GABA_B2_ (Santa, cat. no. qy-1324R, 1:100) subunit, NF200 (Sigma, cat. no. N5389, 1:100), CGRP (Abcam, cat. no. ab81877, 1:100), IB4 (Sigma, cat. no. 2176690, 1:100), GAT1 (Abcam, cat. no. ab277642, 1:100), and IgG control (Abcam) in blocking buffer at 4°C. After being washed three times with blocking buffer, cells were stained with fluorophore-conjugated secondary antibodies (Jackson) for 1 h at RT. As controls for specificity, the primary antibodies were co-incubated with the corresponding immunization peptides, or cells were incubated with the secondary antibody only. All images were collected using a Leica inverted confocal microscope (Leica SP5).

**Chronic pain models.** All surgical procedures were performed under deep anesthesia with an i.p. injection of chloral hydrate (60–80 mg/kg) in accordance with the Animal Care and Ethical Committee of Hebei Medical University and under the International Association for the Study of Pain guidelines. Chronic constriction injury (CCI) was performed as described previously (Sommer and Schafers, 1998). Briefly, rats were anesthetized with an i.p. injection of sodium chloral hydrate (60–80 mg/kg). The right hind leg was shaved and cleaned using 70% ethanol. The sciatic nerve was exposed by blunt dissection at the mid-thigh level, proximal to the sciatic trifurcation. Four nonabsorbable sterile surgical sutures (4–0 chromic gut) were loosely tied around the sciatic nerve with an approximately 1.0-to-1.5-mm interval between the knots. In sham-operated animals, the sciatic nerve was exposed but not manipulated. The skin was sutured, and the animal was transferred to a recovery cage.

**Behavioral tests.** In behavioral tests, animals were habituated to the testing environment for 3 h before behavioral testing. Mechanical and thermal sensitivities were analyzed using the von Frey and Hargreaves methods, respectively, by an operator unaware of the details of the surgical procedure undertaken. The mechanical withdrawal threshold was measured using a set of von Frey filaments (Stoelting Co.) with a calibrated range of bending force (2, 4, 6, 8, and 10 g). Each rat was placed into a plastic cage with a wire mesh bottom. A single filament was applied perpendicularly to the plantar surface of the hind paw five times with an interval of 5 s. Positive response was defined as at least three clear withdrawal responses out of five applications. Filaments were applied in an up-and-down order according to a negative or positive response to determine the hind paw withdrawal threshold. Thermal withdrawal latency was tested using a radiant heat lamp source (PL-200, Taimeng Co.). The intensity of the heat source was maintained at 10 ± 0.1%. Rats were placed individually into plexiglass cubicles placed on a transparent glass surface. The light beam from the radiant heat lamp, located below the glass, was directed at the plantar surface of the hind paw. The time was recorded from the onset of radiant heat stimulation to the withdrawal of the hind paw. Three trials with an interval of 5 min were carried out for each rat, and scores from the three trials were averaged.

**Acute focal application of the AAV2/5 virus to DRG *in vivo*.** AAV2/5-CMV-alpha5 shRNA-GFP (shRNA coding sequence: GCA​CAC​TTC​TCT​ACA​CCA​TGC) and AAV2/5-CMV-GFP viruses were produced by Hanbio Biotechnology Co., Ltd. Virus injections into the L5 DRG were performed as described (Du et al., 2017) under deep anesthesia with an i.p. injection of chloral hydrate (60–80 mg/kg). Briefly, L5 DRGs were exposed by the removal of both spinous and transverse processes of the vertebral bone. A microinjector (Hamilton Co.) was inserted into the ganglion to a depth of 500 μm from the exposed surface. The virus solution (5 μl, ∼8×10^12^ vg/ml of Control shRNA or α5 shRNA) was injected using a constant flow pump at 2 μl/min, and the needle was removed 3 min after the injection was complete. The muscles overlying the spinal cord were loosely sutured together, and the wound was closed. Animals were allowed to recover for at least 4 weeks before the experiments were carried out. Animals developing signs of distress were humanely euthanized. After injection of the AAV2/5 virus into the DRG, the mechanical and thermal sensitivity were analyzed by an operator blind to the viral construct allocation; the von Frey and Hargreaves tests were performed once a week. In some cases, after the behavior testing, some rats were humanely euthanized, and the DRG was extracted for GFP visualization or for quantitative real-time RT-PCR to verify the infection efficiency. In animals subjected to CCI or sham operations, the behavior tests were performed on days 1, 3, 5, 7, 10, and 14.

**Acute focal application of drugs to DRG *in vivo*.** A DRG cannula for focal application of substances to the DRG was implanted as previously described (Du et al., 2017). Briefly, a midline incision was made at the L4–L6 spinal level of adult male rats (Sprague Dawley; 180–200 g), and the L5 was identified at the midpoint of a link between both sides of the iliac crest. A 0.8-mm hole (∼1 mm off the inferior edge of the transverse process) was drilled through the transverse process over the L5 DRG. The approaching of a ganglion was verified by the twitch of the paw. A hooked stainless steel blunt-tip cannula (inner diameter 0.64 mm, length 4 mm) was forced into the hole and connected to a polypropylene tube (inner diameter 0.41 mm, length 4.5 mm). The incision was closed with sutures, and the cannula was firmly fixed in place with dental cement.

For continuous local delivery of drugs, osmotic minipumps (ALZET osmotic pump model 2002) were implanted s.c. in the neck while the cannula tube (ALZET Brain infusion kit 2) connected to the pump was inserted into the DRG as described above. Before the implantation, the infusion drain assembly with the attached osmotic pump was incubated in sterile saline (0.9%) at 37°C for 6 h, after which the minipumps were filled with saline (0.9%) or the α5-GABA_A_ receptor inhibitor, L655708(20 μM). This pump model releases approximately 0.5 μl/h for 14 days. Chronic pain surgical procedures were performed immediately. Intramuscular injection of benzylpenicillin (19 mg/0.1 ml) was given immediately after surgery. Postoperatively, rats were housed individually in plastic cages with sawdust flooring and supplied with water and food *ad libitum*. Animals were left to recover for at least 24 h before the experiments were carried out.

**Statistics**. All data are given as mean ± SEM. Differences between the two groups were assessed using unpaired 2-tailed Student’s *t*-test. Hyperalgesia data in chronic pain models were analyzed using repeated measures ANOVA with the Bonferroni correction. Multiple groups were compared using one-way ANOVA with the Bonferroni correction. The Mann–Whitney test was used for data that failed normality testing. Pearson’s chi-square test was used for comparing the different proportions between the groups. Differences were considered significant at *p* ≤ 0.05. Statistical analyses were performed using OriginPro 9.1 and SPSS. The number of experiments is indicated in each figure; single, double, and triple symbols indicate significant difference from the appropriate control with *p* ≤ 0.05, 0.01, or 0.001, respectively.

## Results

### Expression of Gamma-Aminobutyric Acid–Related Proteins in Dorsal Root Ganglion Neurons of Rats.

**FIGURE 1:**
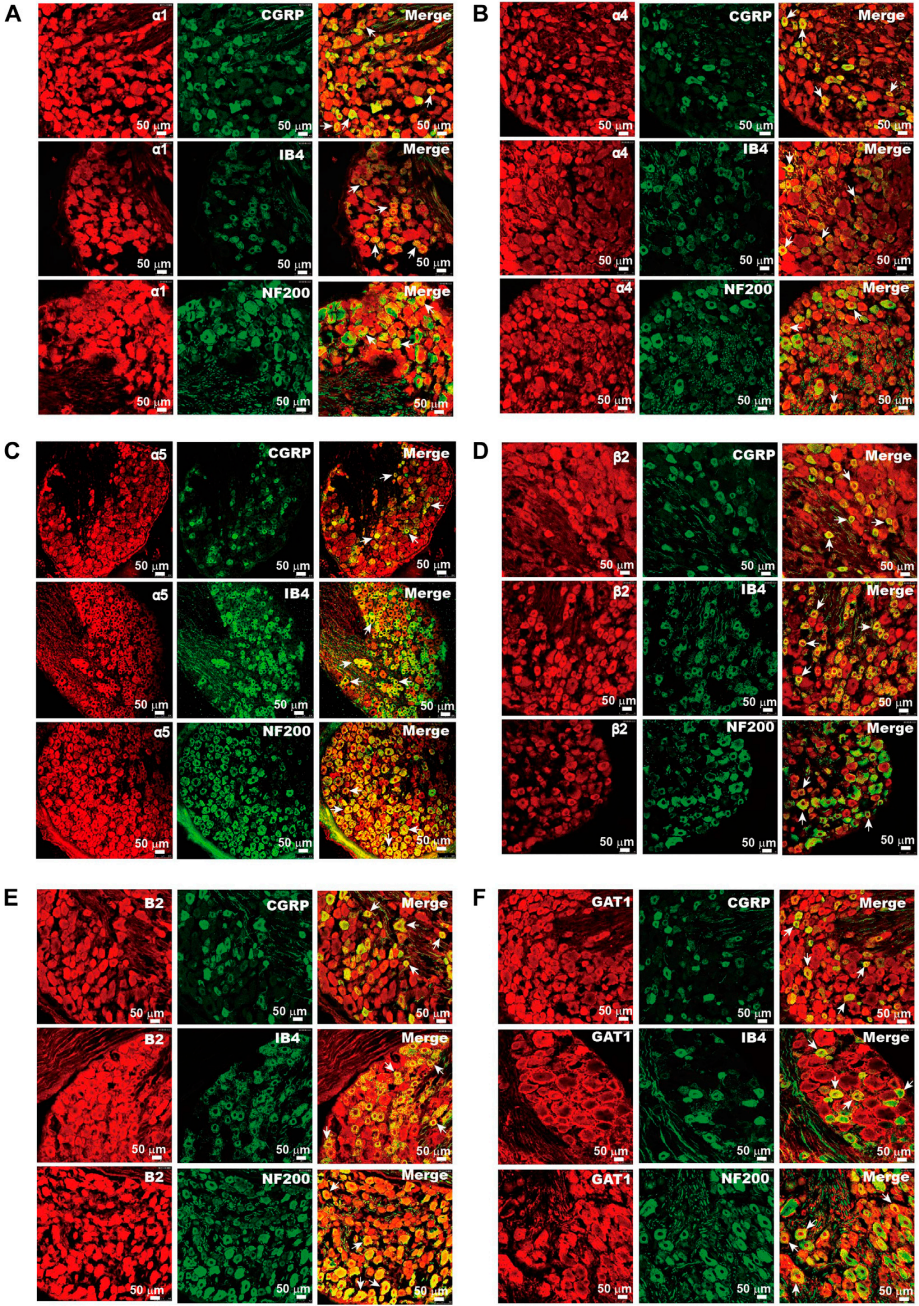
Expression of GABA-related proteins in DRG neurons. Immunofluorescent images of α1 **(Α)**, α4 **(Β)**, α5 **(C)**, and β2 **(D)** of the GABA_A_ receptor, B2 subunit of the GABA_B_ receptor **(E)**, and GAT1 **(F)** in rat DRG neurons. The co-localization of these proteins (red) and sensory neuron markers CGRP, IB4, and NF200 (green) were analyzed. Arrows indicate the representative neurons with co-localization of tested proteins and sensory neuron markers. Micrographs within each panel are of the same magnification; scale bar represents 50 μm.

**FIGURE 2:**
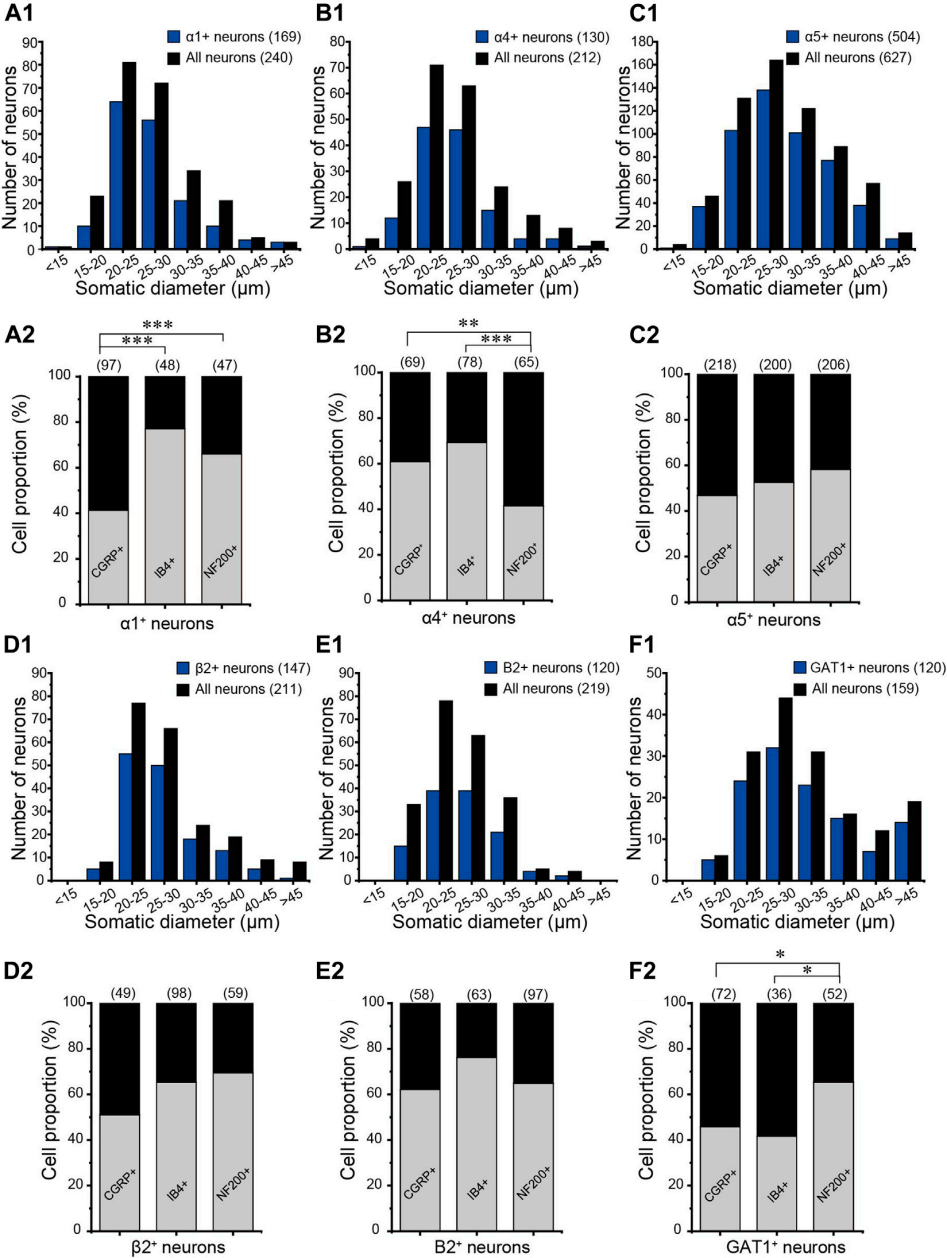
Distributions of GABA-related proteins in DRG neurons. The somatic diameters of neurons positive for α1 **(Α1)**, α4 **(Β1)**, α5 **(C1)**, and β2 **(D1)** of the GABA_A_ receptor, B2 subunit of the GABA_B_ receptor **(E1)**, and GAT1 **(F1)** were measured and plotted as size-distribution histograms. Percentages of CGRP-, IB4-, and NF200-positive neurons in each GABA-related protein–positive DRG neuron were shown in **(A2–F2)**, respectively. *p < 0.05, **p < 0.01, ***p < 0.001 (Pearson’s chisquare test).

Our previous study demonstrated that DRG neurons express mRNAs of key proteins necessary for GABAergic transmission, including multiple GABA_A_ and GABA_B_ receptor subunits, the glutamate decarboxylase isoforms GAD65 and GAD67 (enzymes that decarboxylate glutamate to produce GABA), the vesicular GABA transporter VGAT (which packs GABA into the release vesicles), and the plasma membrane GABA transporters, Gat1–3 (which remove released GABA from the extracellular space). In the present study, we aimed to evaluate possible changes in the DRG’s GABA system that are induced by nerve injury; hence, as a first step, we performed immunohistochemical analysis of expression patterns for key GABA_A_ and GABA_B_ receptors and GAT1 in the rat DRG (Figure 1). The GABA_A_ receptor subunits α1, α4, α5, and β2 (Figures 1A–D) were strongly expressed in the majority of DRG neurons; the expression patterns were partially overlapping with markers of sensory neuron subtypes as follows: CGRP (peptidergic nociceptors), IB4 (nonpeptidergic nociceptors), and NF200 (myelinated fibers) (Figures 1A–D). Likewise, the B2 subunit of GABA_B_ receptors and GABA transporter GAT1 were found abundantly expressed in these subtypes of DRG neurons (Figures 1E,F). The size distributions of neurons expressing each GABA-related protein are analyzed in Figure 2. The size distributions of neurons expressing each GABA-related protein and co-localization with DRG neuron markers are reported in Figure 2. On average, each protein tested was expressed in more than 50% of all DRG neurons and was significantly present in each tested subpopulation (i.e., CGRP^+^, IB4^+^, and NF200^+^; Figures 2A–F). α5, β2, and B2 subunits did not reveal a significant bias toward neurons of a particular size or modality. Interestingly, the α1 subunit was present in a significantly lower proportion of CGRP^+^ neurons than in IB4^+^ neurons and NF200^+^ neurons (*p* = 0.000, *p* = 0.005, respectively; Figure 2A2), while the α4 subunit showed a tendency to localize to neurons with a smaller diameter (Figure 2B1) and was expressed in significantly higher proportions of CGRP^+^ and IB4^+^ neurons than in NF200^+^ neurons (*p* = 0.0025, *p* = 0.001, respectively; Figure 2B2). The expression of GAT1 was significantly higher in NF200^+^ neurons than in CGRP^+^ and IB4^+^ neurons (*p* = 0.03, *p* = 0.031, respectively; Figure 2F2). These results provided further evidence for the existence of peripheral GABAergic signaling within dorsal root ganglia.

### Neuropathic Injury Induces Plastic Changes in Gamma-Aminobutyric Acid–ergic System of Dorsal Root Ganglion Neurons

**FIGURE 3:**
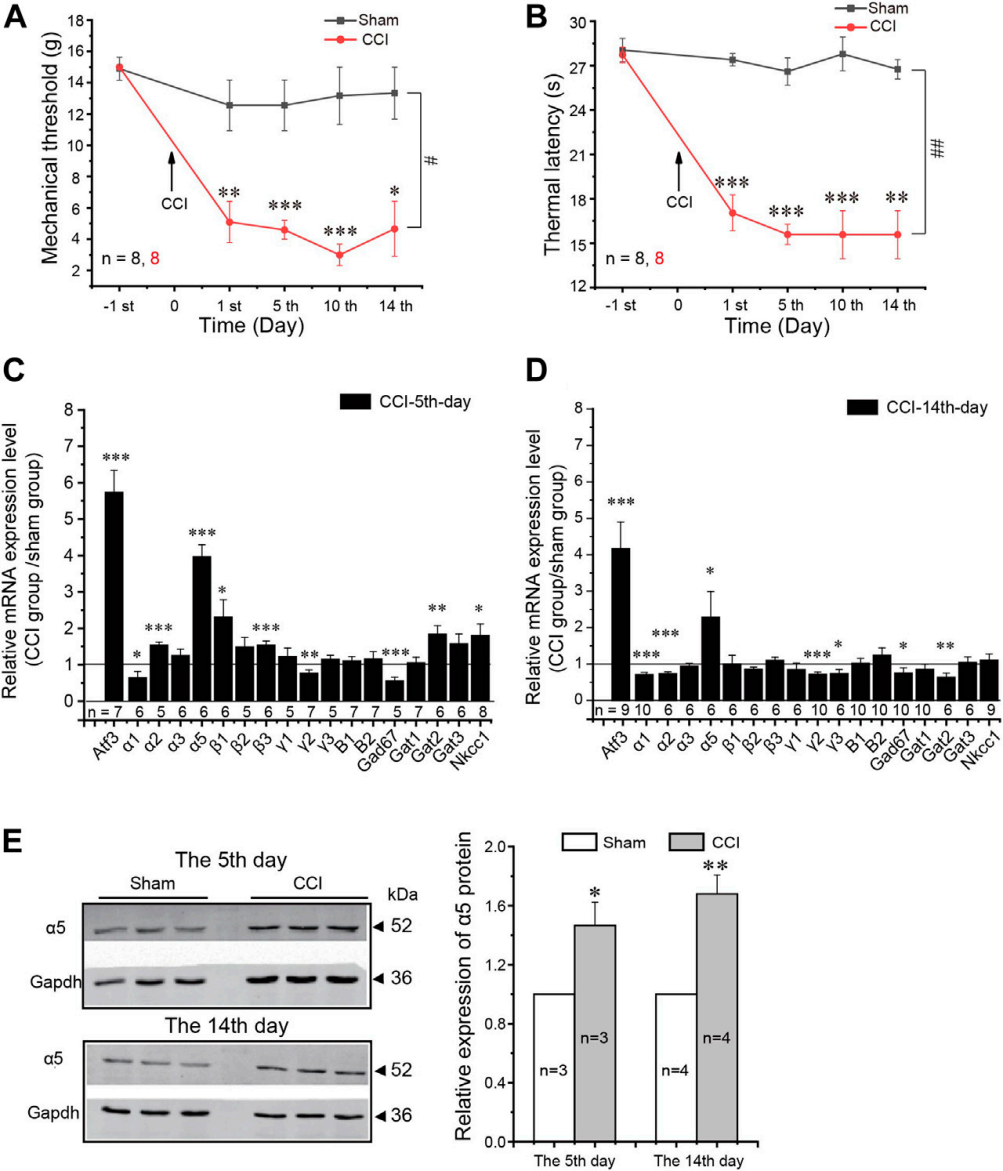
Expression of GABA-related components in the DRG induced by neuropathic pain. **(A,B)** Mechanical threshold **(A)** and thermal latency **(B)** were significantly reduced during the two weeks after CCI surgery in rats. Repeated measures ANOVA with the Bonferroni correction was used for comparison of hyperalgesia measurements between the sham and CCI groups: ^#^ *p* < 0.05, ^##^ *p* < 0.01; one-way ANOVA was used for comparison within the corresponding time point: *p < 0.05. **(C,D)** mRNA relative expression level of indicated GABA-related components in the L4–6 DRGs of rats on the 5th **(C)** and 14th **(D)** days after CCI surgery compared with the sham group. GAPDH was used as the internal reference for the mRNA expression level. One-way ANOVA with the Dunnett *post hoc* test was used for comparison between the tested group and the sham group: *p < 0.05, **p < 0.01, ***p < 0.001. **(E)** Western blot analysis of the α5 subunit of the GABA_A_ receptor in the L4–6 DRGs of rats on the 5th and 14th days after CCI surgery (left, the representative results; right, summary of the changes in the α5 protein level). *p < 0.05, **p < 0.01(unpaired *t*-test). Number of experiments is indicated as *n* in each panel.

Next, we tested if neuropathic injury would induce any change in the expression of GABAergic system components in the DRG. We used chronic constriction injury (CCI) of the sciatic nerve (Bennett and Xie, 1988) in these experiments. CCI induced characteristic, long-lasting hypersensitivity to mechanical and thermal stimuli (Figures 3A,B). RT-PCR analysis of the mRNA of GABA-related genes in ipsilateral L4–L6 DRGs is shown in Figures 3C,D. Consistent with the previous report and with immunohistochemistry data (Figure 1), transcripts for the α1–3 and 5, β1–3, and γ1–3 GABA_A_ subunits, the B1 and B2 GABA_B_ subunits, GAD67, GAT1-3, and NKCC1 were all reliably detectable in the DRG. Compared to the sham group, CCI induced significant increase in the mRNA level of a neuronal injury marker, activating transcription factor 3 (ATF3) (Xu and Yaksh, 2011) at both 5 (Figure 3C) and 14 (Figure 3D) days after injury, confirming neuropathic remodeling of the peripheral nerves. We then analyzed CCI-induced changes in GABA-related genes. At the 5th day after injury, there were bidirectional changes with α1, γ2, and GAD67 being downregulated while α2, α5, β3, GAT2, and NKCC1 upregulated to various extents (Figure 3C). However, by day 14, the general downregulation of GABA receptors and GAD67 became apparent, with α1, α2, γ2, γ3, and GAD67 all being found to be significantly downregulated and (with the exception of α5, see below) all other genes being without a significant change. No obvious changes of GABA_B_ receptors were observed at either time point. Concurrent downregulation of both GABA_A_ subunits and GAD67 indicates the overall weakening of the inhibitory GABAergic system in the DRG throughout neuropathic pain development.

The one striking exception from the overall reduction in the GABA_A_ subunit transcript level was the α5 GABA_A_ subunit, which was consistently and strongly upregulated both at day 5 and at day 14 (Figures 3C,D). The protein levels of α5 were also consistently increased after CCI (as compared to the sham group; Figure 3E).

### Gamma-Aminobutyric Acid–Cl^-^ Currents Evoked From Dorsal Root Ganglion Neurons of Neuropathic Pain Rats

**FIGURE 4:**
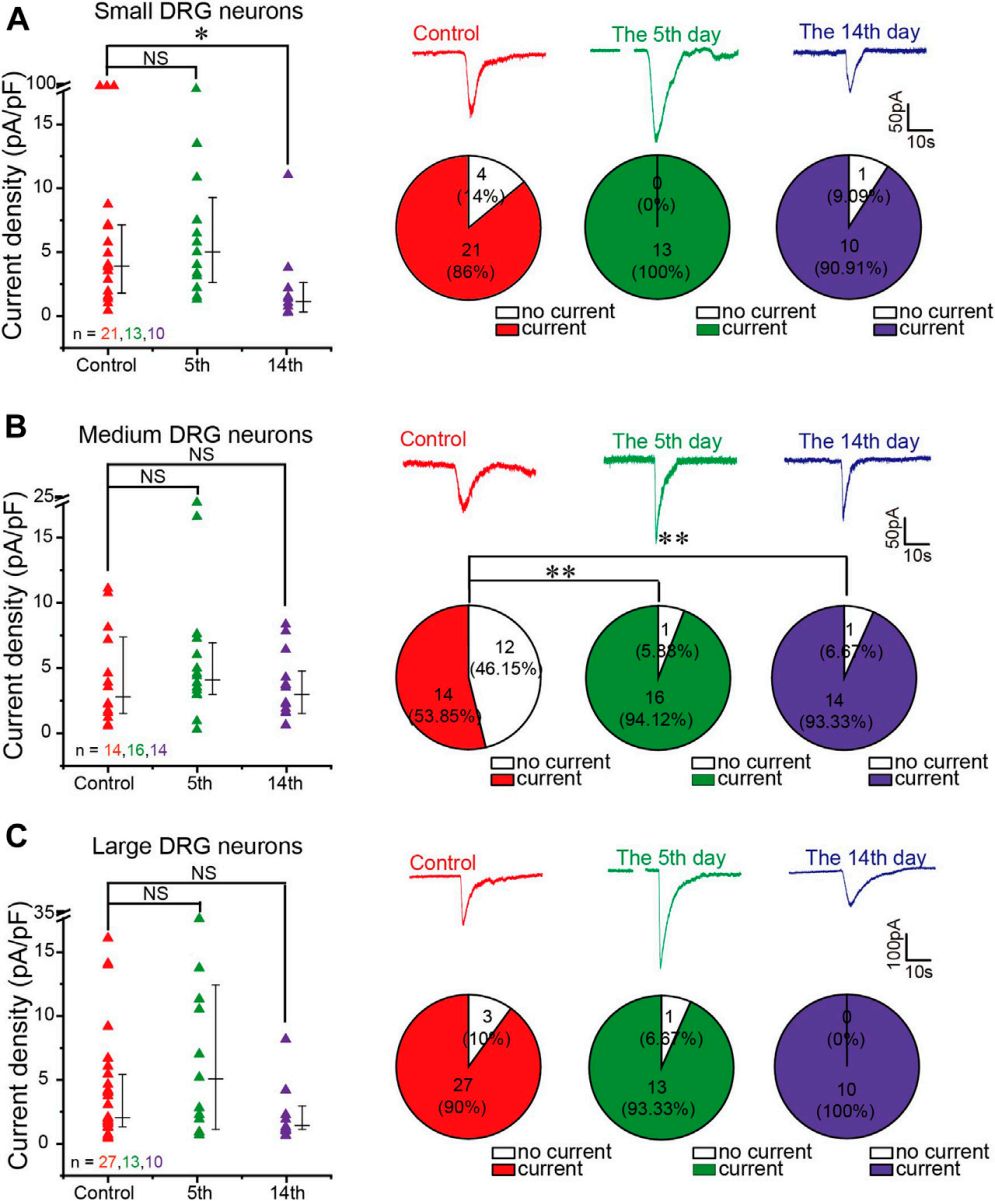
GABA-evoked current from DRG neurons of neuropathic pain rats. **(A–C)** Comparison of GABA (200 μM)-induced currents from isolated and cultured L4–6 DRG neurons from sham-operated and CCI-operated rats on the 5th and 14th days after surgery. Left panels are scatter plots of GABA current densities induced by GABA (200 μM) from small **(A)**, medium **(B)**, and large DRG neurons **(C)** from different groups of rats, respectively, *p < 0.05 (Mann–Whitney test). Right panels are representative traces of GABA currents recorded at a holding potential of −60 mV (top) and the comparison of the proportion of GABA-responding and nonresponding DRG neurons between different groups (bottom) were tested using Pearson’s chi-square test, **p < 0.01. Number of experiments is indicated as *n* in each panel.

To investigate if changes in GABA_A_ subunit expression affected the amplitude of GABA_A_ currents in DRG neurons from neuropathic animals, we performed patch recording of the GABA_A_ current from the acutely dissociated DRG neurons in response to 200 μM GABA from rats subjected to either CCI or a sham procedure. Figure 4 presents the comparison of GABA-induced currents from the DRG neurons of sham-operated animals and animals 5 and 14 days after the CCI. In analyzing these data, we separated the neurons into small (whole-cell capacitance <30 pF, Figure 4A), medium (whole-cell capacitance 30–80 pF, Figure 4B), and large neurons (whole-cell capacitance >80 pF, Figure 4C), representing mostly C, Aδ, and Aβ fibers, respectively (Rola et al., 2003). There was a significant reduction in the GABA_A_ current amplitude in small-diameter DRG neurons 14 days after the surgery, which is in good agreement with the RT-PCR data (Figure 3D). There were no significant changes in the amplitude of GABA responses in the other DRG groups; there was a tendency toward larger responses at the 5th day after the CCI in large neurons, and at day 14, the currents were somewhat reduced in both medium and large neurons, but these tendencies did not reach significance.

Interestingly, in the small and large neuron subpopulations, the majority of neurons (over 80%) responded to GABA with sizable inward currents (both in control and in CCI animals) (Figures 4A,C). In contrast, only ∼50% of medium neurons from the sham controls responded to GABA (Figure 4B), but the proportion of responding neurons was significantly higher after the CCI (∼94 and ∼93% at 5 and 14 days after the CCI, respectively; Figure 4B). One hypothesis is that the increase in the proportion of GABA-responding medium neurons may be caused by CCI-induced overexpression of the α5 GABA_A_ subunit (Figures 3C–E).

### *In Vivo* Silencing of the α5 Gamma-Aminobutyric Acid_A_ Subunit Alleviates Neuropathic Pain

**FIGURE 5:**
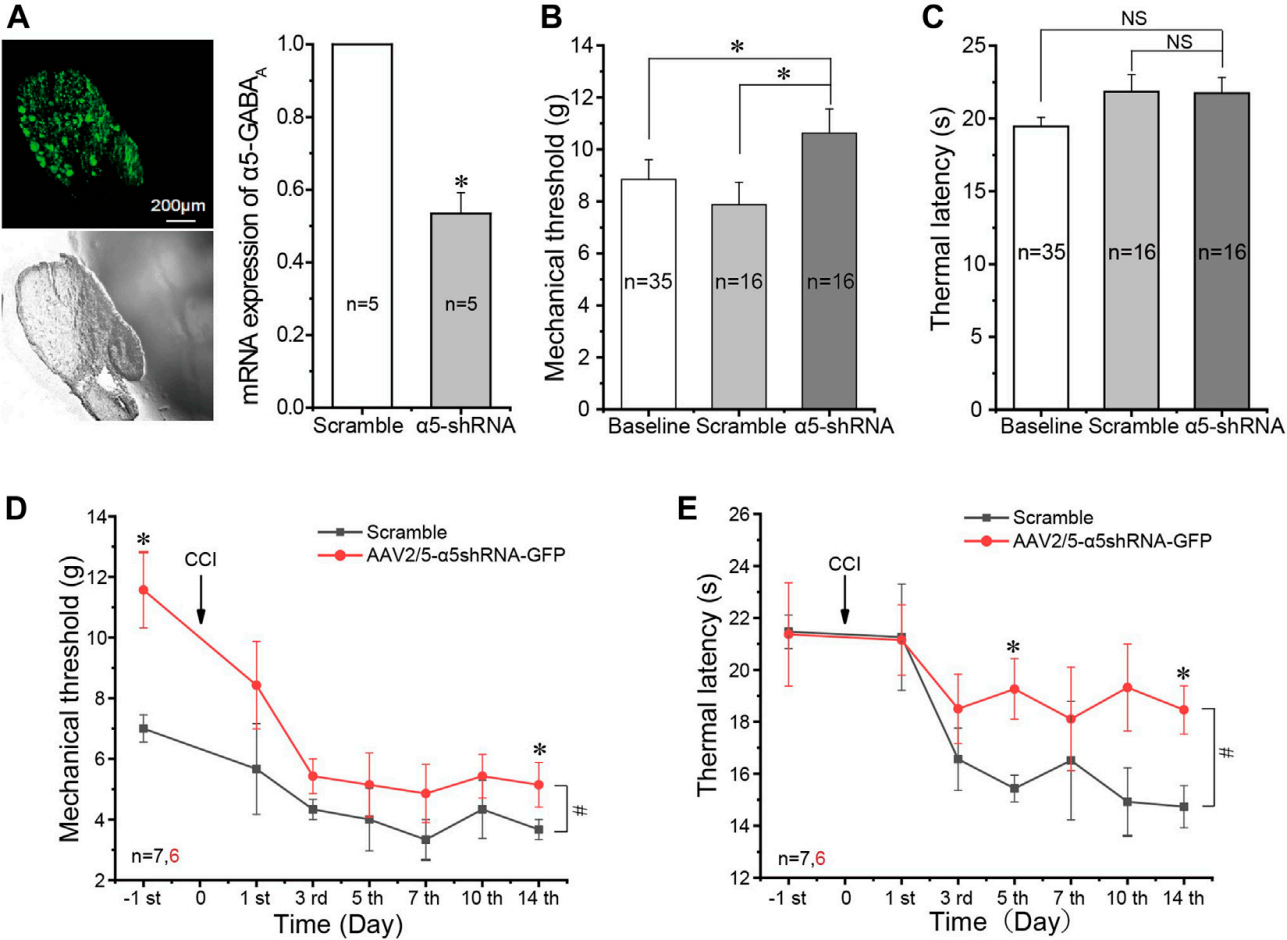
*In vivo* silencing of the α5 GABA_A_ subunit alleviates neuropathic pain. (**A)** Injection of AAV2/5-α5 shRNA-GFP into the L5 DRG of rats *in vivo* produced strong fluorescence in the DRG and significantly reduced mRNA expression of the α5 subunit in the DRG (*n* = 5), *p < 0.05 (unpaired *t* test). **(B)** Injection of AAV2/5-α5 shRNA-GFP into the L5 DRG of rats significantly increased the mechanical threshold as compared to the baseline threshold and injected scrambled RNA, *p < 0.05 (one-way ANOVA with the LSD *post hoc* test). **(C)** No significant change of thermal latency. **(D,E)** Injection of AAV2/5-α5 shRNA-GFP into the L5 DRG significantly alleviated mechanical **(D)** and thermal **(E)** hyperalgesia produced by CCI. Repeated measures ANOVA with the Bonferroni correction was used for comparison of hyperalgesia measurements between the groups, ^#^ *p* < 0.05; one-way ANOVA was used for comparison within the corresponding time point: *p < 0.05. Number of experiments is indicated as *n* in each panel.

Our data thus far demonstrated upregulation of the α5 GABA_A_ subunit and a concurrent increase in the proportion of GABA-responsive medium-size neurons during the development of CCI-induced neuropathic pain. Thus, we hypothesized that the α5 subunit may play a unique pro-algesic role in the sensory nerves. We then set out to investigate how an *in vivo* silencing of this GABA_A_ subunit in DRG neurons might affect the development of the neuropathic pain. We used an shRNA knockdown approach whereby the AAV2/5-shRNA-GFP virus against the α5 subunit was designed and directly injected into the L5 DRG of rats (see *Materials and Methods*). The control rats were injected with AAV2/5 expressing scrambled shRNA. GFP fluorescence was readily detectable in the L5 DRG of rats at 4 weeks after the virus injection, and the mRNA of the α5 subunit was knocked down by 47.6 ± 0.06% (*n* = 5) in knockdown rats (Figure 5A). Noticeably, there was a small but significant decrease in basal mechanical sensitivity (increased withdrawal threshold, see *Materials and Methods*) in rats injected with AAV2/5-shRNA-GFP against the α5 subunit (10.63 ± 0.93 g, *n* = 16), as compared with the scrambled shRNA–injected control rats (7.88 ± 0.86 g, *n* = 16; Figure 5B). But there was no significant difference in basal thermal sensitivity (withdrawal latency, see *Materials and Methods*) between the two groups (Figure 5C).

**FIGURE 6:**
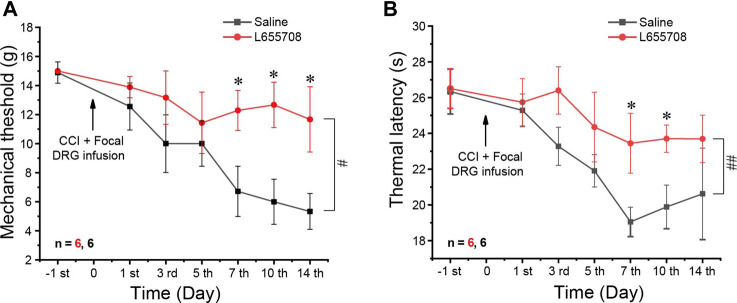
Focal infusion of the α5 GABAA subunit antagonist alleviates neuropathic pain *in vivo*. Focal DRG application of L655708 (20 μM, ∼0.5 μl/h) using an osmotic minipump significantly alleviated mechanical **(A)** and thermal **(B)** hyperalgesia produced by the CCI. Repeated measures ANOVA with the Bonferroni correction was used for comparison of hyperalgesia measurements between the groups, ^#^ *p* < 0.05, ^##^ *p* < 0.01; one-way ANOVA was used for comparison within the corresponding time point: *p < 0.05. Number of experiments is indicated as *n* in each panel.

We then performed CCI on animals that were pre-injected 4 weeks before either with AAV2/5-shRNA-GFP against the α5 subunit or with the AAV2/5 expressing scrambled shRNA. α5 knockdown animals developed milder mechanical and thermal hyperalgesia than the scrambled shRNA controls (Figures 5D,E). The alleviation of thermal hyperalgesia was more prominent (Figure 5E), while mechanical hyperalgesia was only borderline reduced; only at day 14 after the CCI did the difference reach significance (Figure 5D). Taken together, the data presented in Figure 5 suggest that unlike other GABA_A_ receptor subunits, the α5 subunit plays a pro-algesic role in the DRG. To further test this, we tried to continuously apply the specific α5 subunit antagonist L665708 (Quirk et al., 1996; Delgado-Lezama et al., 2013) to the DRG using an implanted osmotic minipump (see *Materials and Method*). The minipumps were installed at the time of the CCI surgery and slowly released L665708 (20 μM) at a speed of 0.5 μl/h for approximately 14 days. As expected, L665708 significantly reduced both mechanical (Figure 6A) and thermal hyperalgesia (Figure 6B) induced by CCI surgery. These results suggested that the α5 subunit can be a therapeutic target for chronic neuropathic pain.

## Discussion

In this study, we report the expression of multiple GABA_A_ and GABA_B_ receptor subunits, as well as GAD67, GAT1-3, and NKCC1, in the rat DRG. We found that neuropathic injury induced general downregulation of most GABA_A_ receptor subunits and GAD67 in the whole DRG tissue and concomitant reduction in GABA-induced currents in small-diameter DRG neurons. Uniquely among the GABA_A_ subunits tested, the α5 subunit was strongly and consistently upregulated. We also noted that the proportion of GABA-responding medium-size DRG neurons was also increased following neuropathic injury. Finally, selective knockdown or blocking of the α5 GABA_A_ subunit in the DRG *in vivo* produced moderate alleviation of neuropathic hyperalgesia. These findings suggest that while neuropathic injury is associated with general weakening of the GABAergic inhibitory system in the DRG, an elevated α5 GABA_A_ subunit might play an unexpected excitatory and pro-algesic role in the peripheral sensory system.

GABA_A_ receptors are heteropentameric ligand-gated chloride channels. Several subunits have been cloned to constitute a functional GABA_A_ receptor, including *α* (1–6), *β* (1–3), *γ* (1–3), *ε*, *δ*, *θ*, *π*, and *ρ* (1–3) (Olsen and Sieghart, 2008). Our study and earlier reports (Lee et al., 2012; Lee and Gold, 2012; Zhu et al., 2012; Obradovic et al., 2015; Loeza-Alcocer et al., 2019; Wilke et al., 2020) demonstrated that DRG neurons express multiple subunits of the GABA_A_ receptor. It is generally accepted that GABA_A_ receptors expressed in DRG neurons would traffic to afferent terminals and would be activated by GABA released in the spinal cord to produce an inhibitory PAD (Price et al., 2009; Wilke et al., 2020). Yet, activation of GABA_A_ receptors in DRG neuron somata could depolarize the membrane potential and cause action potential failure to propagate through the *t*-junction (Du et al., 2017). Thus, normally, depolarization of either afferent terminals or the somatic/perisomatic compartment of the afferent fiber would result in an inhibition of nerve transmission. In this context, weakening of the peripheral GABAergic system, reported here, is consistent with a general increase in the peripheral nerve excitability and peripheral nociceptive transmission associated with chronic pain development. Our data are also consistent with the widely acknowledged neuropathic weakening of spinal GABAergic inhibitory circuits (Braz et al., 2014; Duan et al., 2014; Mendell, 2014; Peirs et al., 2015; Zhang et al., 2018).

Nevertheless, upregulation of NKCC1, which facilitates the accumulation of intracellular Cl^−^, drives excitation of the membrane potential in afferent terminals and contributes to injury- or inflammation-induced pain (Zhu et al., 2012). The NKCC1 function could be upregulated by phosphorylation (Darman and Forbush, 2002; Flemmer et al., 2002) or enhancement of its expression and both phenomena were reported in neuropathic nerves (Hasbargen et al., 2010; Chen et al., 2014; Modol et al., 2014). We did not examine phosphorylation of NKCC1 in this study, but we found that the NKCC1 mRNA level was increased on day 5 post-CCI (there was no significant change at day 14). This may suggest that GABAergic inhibition could have been diminished or even inverted in some DRG neurons which have accumulated excessive Cl^–^ levels. The proportion of medium-size DRG neurons responding to GABA was increased on both days 5 and 14 post-CCI. These findings may suggest that a subpopulation of Aδ DRG neurons, after neuropathic injury there, could have acquired a *de novo* sensitivity to GABA. We further hypothesize that this acquired sensitivity is driven by the upregulation of the α5 GABA_A_ subunit and that it might be excitatory. Consistent with this hypothesis, *in vivo* downregulation of the α5 subunit produced moderate alleviation of the CCI-induced hyperalgesia. Future studies are necessary to put these hypotheses to the test.

Changes in GABA_A_ receptor subunit expression and function in peripheral nerves under pathologic chronic pain conditions were indeed demonstrated. For instance, Michael Gold’s group demonstrated that persistent inflammation increased GABA_A_ currents in rat DRG neurons through upregulating the high-affinity GABA_A_ receptor subunits *δ* and *ρ* (Lee et al., 2012; Lee and Gold, 2012). GABA_A_ receptors containing the α5 subunit are another type of high-affinity GABA_A_ receptor expressed in DRG neurons of rats (Bravo-Hernandez et al., 2014). Another study reported that the α5 subunit is mainly present in small-to-medium–size nonpeptidergic neurons. They provided evidence that α5 subunit–containing GABA_A_ receptors mediated a tonic state of excitability of primary afferents in the turtle (Loeza-Alcocer et al., 2013). Furthermore, a previous study showed that formalin injection enhanced α5 subunit–containing GABA_A_ receptor expression in the DRG, and the α5-GABA_A_ receptor antagonist L655708 prevented and reversed long-lasting formalin-induced hypersensitivity (Bravo-Hernandez et al., 2016). Taken together, our data, together with previous results, indicate that α5 subunit–containing GABA_A_ receptors indeed have pronociceptive effects in the periphery.

On the contrary, the α2 subunit of GABA_A_ receptors is clearly antinociceptive (Obradovic et al., 2015). Thus, crush injury of the sciatic nerve induced significant downregulation of the α2 subunit in the rat DRG; selective downregulation of α2 subunit expression in the DRG significantly worsened mechanical and thermal hypersensitivity in crush-injured animals and caused development of significant mechanical and thermal hypersensitivity in sham animals. Importantly, in our study, the α2 subunit was found to be significantly downregulated at day 14 (but not at day 5) post-CCI. The opposing effect of two similar Cl^−^ channel subunits in sensory nerves is intriguing and requires further investigation. The following needs to be taken into consideration: i) these subunits may be expressed in different subpopulations of DRG neurons; moreover, α5 subunits may be *de novo* expressed in some injured fibers (see above). ii) These subunits may distribute to different locations in DRG neurons (i.e., peripheral terminals, somata, stems, dorsal roots, and central terminals) with potentially diverse local E_Cl_ of the membrane. iii) In contrast to α2, α5 is a high-affinity GABA_A_ subunit (Farrant and Nusser, 2005; Delgado-Lezama et al., 2013). iv) There may be other biophysical differences between neurons expressing α2 and α5 subunits (e.g., the repertoire and density of voltage-gated Na^+^ channels), which may confer a differential impact of Cl^−^ channel activity on excitability. Thus, opposing effects of channels belonging to the same family may not be entirely surprising.

Interestingly, the effects of α5 knockdown on thermal and mechanical sensitivities were different: the basal mechanical sensitivity was reduced, while the basal heat sensitivity was not affected (Figures 5C,D). Yet, the knockdown had a much more prominent effect on thermal hyperalgesia induced by CCI than on mechanical hyperalgesia (Figures 3D,E). The reasons for such an effect pattern are yet to be elucidated by further experiments.

In summary, here we report long-lasting plastic changes in the peripheral GABAergic system induced by neuropathic injury. Furthermore, we show that the α5 GABA_A_ subunit may have a unique pro-algesic role and might represent a new therapeutic target for pain relief.

## Data Availability

The original contributions presented in the study are included in the article/Supplementary Material; further inquiries can be directed to the corresponding authors.
