# Supplementary material for: Neuropathic Injury–Induced Plasticity of GABAergic System in Peripheral Sensory Ganglia
Source: Front Pharmacol. 2021 Jul 27;12:702218. doi: 10.3389/fphar.2021.702218 (PMC8354334; doi:10.3389/fphar.2021.702218)
Supplement: Supplementary file 1 [file Table1.docx]

## Supplemental Table 1. List of primers used in RT-PCR experiments

| **Gene** | **Primer** | **Length**  **(bp)** |
| --- | --- | --- |
| ATF3 | F 5’-AGTCAGTCACCATCAACAACAGA-3’  R 5’-GCATTCACACTCTCCAGTTTCTC-3’ | 190 |
| α1 | F 5’-TGTCTTTGGAGTGACGACCG-3’  R 5’-ATCCCACGCATACCCTCTCT-3’ | 187 |
| α2 | F 5’-AGGTTGCTCCTGATGGCTCTA-3’  R 5’-TCTCGCTGGCACCGATTCT-3’ | 227 |
| α3 | F 5’-CTCCCAGTGCTTCTTCAACTCC-3’  R 5’-CTGCCACTATTATCTACTGTTTGCG-3’ | 231 |
| α5 | F 5’-CAAAACGCTCCTTGTCTTCTG-3’  R 5’-TGTGATGTTGTCATTGGTCTCA-3’ | 106 |
| β1 | F 5’-TGTGTTCGTGTTCCTGGCTCTACT-3’  R 5’-GCATCAACCTGGACTTTGTTCATC-3’ | 147 |
| β2 | F 5’-TGACCACAATCAATACCCATCT-3’  R 5’-ACAAAGACAAAGCACCCCATTA-3’ | 94 |
| β3 | F 5’-CTGTACGGGCTCAGGATCAC-3’  R 5’-GGGAGCTCGATCCTTTCCAC-3’ | 179 |
| γ1 | F 5’-AGACGGATGGGCTATTTCACAA-3’  R 5’-ATACCCAGGGATGTTCTAGCAGG-3’ | 119 |
| γ2 | F 5’-CGCAGTTCTGTTGAAGTGGG-3’  R 5’-CAGGGAATGTAGGTCTGGATGG-3’ | 179 |
| γ3 | F 5’-TGGTCTATTGGGTTGGATACCT-3’  R 5’-CACTACTTGTCTGGGGATGATG-3’ | 104 |
| B1 | F 5’-TCATCGGGTGGTATGCTGAC-3’  R 5’-GTTGGAAATGCTTCGGGTGT-3’ | 146 |
| B2 | F 5’-CTGGGCAAAATCATCCTCAAT-3’  R 5’-GACCTTCACCTCTCTGCTGTCT-3’ | 135 |
| GAD67 | F 5’-CTTGTGAGTGCCTTCAGGGAG-3’  R 5’-CTTGCGGACATAGTTGAGGAGTA-3’ | 204 |
| GAT1 | F 5’-GATGGACTGGAAAGGTGGTCTA-3’  R 5’-CATTGTTGTGGAAAGAGTTGTAGC-3’ | 246 |
| GAT2 | F 5’-AGCGCTGGTGGACATGTATC-3’  R 5’-TGTGAGCATAATGAGCCCGA-3’ | 109 |
| GAT3 | F 5’-CTACCCCAAGGCTGTCACTATG-3’  R 5’-GGCTCTCCACACACACAAACT-3’ | 110 |
| NKCC1 | F5’-GCATTCAATCCGTCTTTCTGG-3’  R5’-GGCCACAGATCATTAAACCAAC-3’ | 138 |
| GAPDH | F 5’-GACATGCCGCCTGGAGAAAC-3’  R 5’-AGCCCAGGATGCCCTTTAGT-3’ | 92 |
